# Hemolysis of Human Erythrocytes by Argovit™ AgNPs from Healthy and Diabetic Donors: An In Vitro Study

**DOI:** 10.3390/ma14112792

**Published:** 2021-05-24

**Authors:** Roberto Luna-Vázquez-Gómez, María Evarista Arellano-García, Juan Carlos García-Ramos, Patricia Radilla-Chávez, David Sergio Salas-Vargas, Francisco Casillas-Figueroa, Balam Ruiz-Ruiz, Nina Bogdanchikova, Alexey Pestryakov

**Affiliations:** 1 Facultad de Ciencias, Universidad Autónoma de Baja California (UABC), Ensenada 22860, Baja California, Mexico; rluna@uabc.edu.mx (R.L.-V.-G.); casillas.francisco@uabc.edu.mx (F.C.-F.); 2 Escuela de Ciencias de la Salud, Unidad Valle Dorado, Ensenada 22890, Baja California, Mexico; patyradilla@uabc.edu.mx (P.R.-C.); salasd@uabc.edu.mx (D.S.S.-V.); 3 Departamento de Ciencias de la Salud, Unidad Regional Los Mochis, Universidad Autónoma de Occidente, Los Mochis 81223, Sinaloa, Mexico; balam.ruiz@uadeo.mx; 4 Nanoscience and Nanotechnology Center (CNyN), National Autonomous University of Mexico (UNAM), Mexico City 58089, Distrito Federal, Mexico; nina@cnyn.unam.mx; 5 Research School of Chemistry & Applied Biomedical Sciences, Tomsk Polytechnic University, 634050 Tomsk, Russia

**Keywords:** in vitro hemolysis, silver nanoparticles, diabetic erythrocytes

## Abstract

The use of nanomaterials is becoming increasingly widespread, leading to substantial research focused on nanomedicine. Nevertheless, the lack of complete toxicity profiles limits nanomaterials’ uses, despite their remarkable diagnostic and therapeutic results on in vitro and in vivo models. Silver nanoparticles (AgNPs), particularly Argovit™, have shown microbicidal, virucidal, and antitumoral effects. Among the first-line toxicity tests is the hemolysis assay. Here, the hemolytic effect of Argovit™ AgNPs on erythrocytes from one healthy donor (HDE) and one diabetic donor (DDE) is evaluated by the hemolysis assay against AgNO_3_. The results showed that Argovit™, in concentrations ≤24 µg/mL of metallic silver, did not show a hemolytic effect on the HDE or DDE. On the contrary, AgNO_3_ at the same concentration of silver ions produces more than 10% hemolysis in both the erythrocyte types. In all the experimental conditions assessed, the DDE was shown to be more prone to hemolysis than the HDE elicited by Ag^+^ ions or AgNPs, but much more evident with Ag^+^ ions. The results show that Argovit™ is the least hemolytic compared with the other twenty-two AgNP formulations previously reported, probably due to the polymer mass used to stabilize the Argovit™ formulation. The results obtained provide relevant information that contributes to obtaining a comprehensive toxicological profile to design safe and effective AgNP formulations.

## 1. Introduction

Nanomaterials for biological applications have been successfully developed in the last decades, e.g., theranostics, drug delivery, imaging, and sensors [1]. Their unique physicochemical properties, such as high surface-to-volume ratio, high porosity, and fine-tune modulation of properties at the atomic level, provide them with a reactivity enhancement compared with bulk materials. The aforementioned characteristics make nanoparticles and nanomaterials attractive for pharmaceutical, food, cosmetics, energy, and construction industries among others. Nevertheless, these unique properties could also produce adverse effects on human health and the environment, modifying the rate and ratio of the processes previously described for bulk materials. So, it is necessary to identify and evaluate the risks and factors associated with the use of any nanomaterial [2].

Silver nanoparticles (AgNPs) are among the most used nanomaterials in the biomedical field due to their microbicidal effect, despite the lack of evidence detailing a clear mechanism of action [3,4]. Most of the reports suggest the silver ion release as the main effector of the biological response [5,6,7]; however, some suggest that the complete nanoparticle, and not the released ions, is responsible for the observed effect [8,9]. Most of the publications report no selectivity on the cytotoxic and genotoxic damage produced by AgNPs formulations, mainly those uncoated or unstable coated AgNP formulations [10,11,12,13,14,15,16]. Nonetheless, including a coating agent that provides greater stability to the AgNPs formulation, such as polyvinylpyrrolidone (PVP), can improve cytotoxic selectivity [8,9].

One of these PVP–AgNPs formulations, known as Argovit™, has been studied in several in vitro and in vivo models [17,18,19,20,21], showing remarkable results without any cytotoxic/genotoxic effects [22,23] observed on the non-transformed or non-pathogenic cells. The effective concentrations employed attributed to the high stability provided by the high amount of PVP used to obtain this AgNP formulation. Besides, biodistribution studies on rats show that 98% of this formulation was excreted in the feces [24,25,26], a result that confirms its low toxicity. To better understand the toxicological profile, an evaluation of the hemolytic effect of this formulation on human erythrocytes is indispensable.

**Figure 1:**
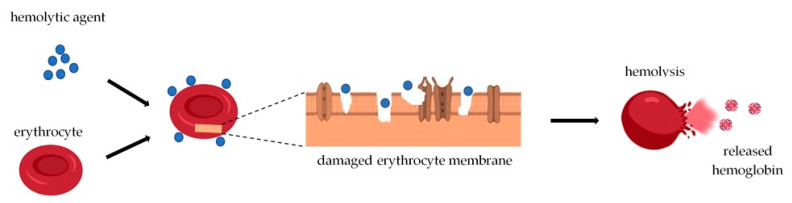
Basic mechanism of hemolysis.

The Scientific Committee for Consumer Safety (SCCS) established the hemolysis test as a necessary test for human consumption product approval [27]. According to the American Society for Testing and Materials (ASTM), less than 5% hemolysis is considered null [28]. Above this limit and up to 10% is assumed as low, and beyond 10% is perceived as marked hemolysis. Hemolysis represents the rupture or alteration of the integrity of the red blood cell membrane, causing the release of hemoglobin [29]. Figure 1 shows the basic mechanism of hemolysis.

In the physiological environment, the red cell membrane rupture allows the cell contents to escape into the plasma. The most abundant cytosolic content of red blood cells is hemoglobin, which comprises 97% of the red blood cells. Hemoglobin is specialized in the transport of blood gases in animals; therefore, significant hemolysis compromises all tissue functionality [30]. Numerous studies describe the hemolysis produced by AgNP formulations in erythrocytes from healthy donors (HDE) [31,32,33,34,35,36,37,38,39,40,41,42,43,44,45,46]. Many AgNPs have been used to treat diabetic foot ulcers and other diabetic-related wounds [47,48,49], including Argovit™ [50]. However, no studies have been found based on experiments with erythrocytes from diabetic donors (DDE). DDE and HDE show different susceptibilities to hemolysis due to the structural changes related to osmotic stress and greater membrane fragility that hyperglycemia induces in red blood cells [51,52]. Therefore, this work aims to identify the hemolytic capacity of Argovit™ and compare it with the hemolysis produced by Ag^+^ from AgNO_3_ to improve our knowledge about both agents toxicological response in DDE and HDE. Among the published works on hemolysis by AgNPs, the results are expressed in concentrations of complete AgNPs without indicating the content of metallic silver. This makes the results interpretation difficult because cytotoxicity (e.g., hemolysis) has been attributed mainly to the release of silver ions, which is why in this work both the concentration of AgNPs, and their Ag content are presented.

## 2. Materials and Methods

### 2.1. Characterization of AgNPs Formulation Argovit™

Argovit™ was provided by Dr. Vasily Burmistrov from Vector-Vita Scientific and Production Center (Novosibirsk, Russia). Argovit™ is a stable water suspension with final concentration of 200 mg/mL of AgNPs (20% *w*/*w*). The metallic silver content (1.2% *w*/*w*) is stabilized with polyvinylpyrrolidone (PVP 12.6 ± 2.7 kDa), which represents 18.8% of the formulation’s total weight. The other 80% of the weight corresponds to distilled water. AgNPs have a spherical shape with a size distribution from 1 to 90 nm in diameter, with an average of 35 ± 12 nm. The hydrodynamic diameter is 70 nm, the ζ potential is −15 mV, and it has a plasmonic resonance at 420 nm [53]. Argovit™ AgNPs have been widely used in medical, veterinary, and industrial applications and have the corresponding certificates of use [21,50].

### 2.2. Preparation of Solutions

Argovit^TM^ AgNPs: at 12, 24 and 48 µg/mL of Ag content at which the erythrocytes were exposed, corresponding to 200, 400 and 800 µg/mL of AgNPs (Table 1).

AgNO_3_: Sigma-Aldrich 2091-39-25G (St. Louis, MO, USA), selected as a source of Ag^+^ ions due to its high water solubility at 12, 24 and 48 µg/mL of Ag content at which the erythrocytes were exposed (Table 1).

Positive control: Triton X-100 at 20% *v*/*v* solution was prepared as a positive control.

Negative control: A standard PBS solution adjusted to pH = 7.4.

### 2.3. Preparation of Erythrocyte Suspensions

Venous blood (25 mL) was drawn by vacuum phlebotomy followed by centrifugation at 500× *g* for five minutes. Plasma was discarded and red blood cells were washed three times with sterile NaCl 150 mM physiological solution. A final wash was carried out with PBS buffer solution at pH = 7.4 under the same conditions. The supernatant was replaced with new PBS solution pH = 7.4. For the preparation of the experiments, 1 mL of the erythrocyte pack from the before obtained suspension was diluted in 49 mL of PBS at pH 7.4 achieving a 1:50 erythrocyte stock suspension.

### 2.4. Hemolysis Test

Three independent hemolysis experiments were performed, each in triplicate (*n* = 3) in 1.5 mL conical bottom tubes, with AgNPs Argovit™ and AgNO_3_ at concentrations of 12, 24 and 48 µg/mL of silver. Additionally, negative control (PBS) and positive control (TritonX-100,) (Thermo Fisher Scientific Inc., Rockford, IL, USA) were used. The assay was set up to achieve a final volume of 1000 µL in each trial. This was accomplished by adding 50 µL of the test agent to 950 µL of the 1:50 red cell stock dilution. All tubes were incubated simultaneously at 37 °C for 2 h shaking them by gentle inversion once every half hour. Then the tubes were centrifuged at 500× *g* for 5 min and 100 µL of the supernatant was transferred to 96-well plates to obtain absorbance readings at 450 nm in a 96-well ControLab EliRead spectrophotometer (RT-21007) (EliRead, KontroLab, Italy). The hemolysis percentages were calculated using the following formula:

$$\%\;H=100*[\frac{\mathrm{Ap}-\mathrm{Ac}{\left(-\right)}_{\mathrm{ind}}}{\mathrm{Ac}\left(+\right)-\mathrm{Ac}\left(-\right)}$$

where,

**% H** = percentage of hemolysis

**Ap** = absorbance of sample

**Ac**(**−**)**_ind_** = absorbance of negative control with erythrocytes in each sample

**Ac**(**+**) = absorbance of positive control

**Ac**(**−**) = absorbance of negative control with erythrocytes

### 2.5. Donors

Healthy donor erythrocytes were obtained from a 36-year-old male normocytic individual weighing 70 kg, a body mass index of 23.7 with hematocrit of 47.4%, and a fasting glucose of 90 mg/dL. No apparent pathological data in the past 10 years is referred by this individual.

Diabetic donor erythrocytes were obtained from a 35-year-old male normocytic individual weighing 139 kg, a body mass index of 38.5, with hematocrit of 45.1%, a fasting glucose of 140 mg/dL and glycosylated hemoglobin (Hb A1c) of 10%. The individual was diagnosed in 2016 with type 2 diabetes (T2D). Subcutaneous insulin Tresiba™ at 30 units per day and Humalog™ 10 units per day were prescribed and administered ever since T2D was diagnosed. Other than obesity, no comorbidities nor complications are referred by this individual.

### 2.6. Statistical Analysis

After verifying homoscedasticity with the Bartlett test, a multifactor analysis of variance was performed to detect statistical differences in hemolysis data with n = 9 in three independent experiments with three replicates. A Tukey’s post-hoc test was made with Statistica V.13.3 1984–2017, (TIBCO Software Inc., Palo Alto, CA, USA) (see Supplementary Materials Table S1), and graphs generated with GraphPad Prism 8.1.0.

## 3. Results and Discussion

**Figure 2:**
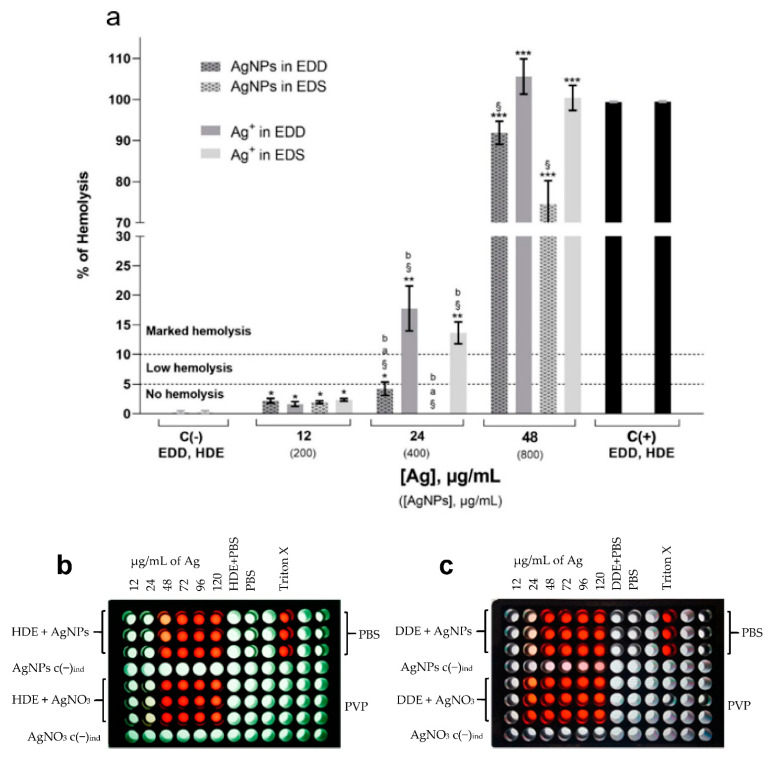
(**a**) Hemolysis in diabetic donor erythrocytes (DDE) and healthy donor erythrocytes (HDE) induced by AgNPs and Ag^+^, PBS negative controls C(−) and Triton X-100 C(+). Dotted horizontal lines separate the limits of no hemolysis (<5%), low hemolysis (5–10%), and marked hemolysis (>10%). The average of PBS negative controls C(−) and Triton X-100 positive controls C(+) are presented. The standard error of the mean (SEM) is shown in each bar. *p*-value is indicated on the top of each bar, * (*p* ≤ 0.05), ** (*p* ≤ 0.01), and *** (*p* ≤ 0.001) compared with the negative control. ^§^ *p* ≤ 0.01 compared to positive control. ^a^ *p* ≤ 0.01 comparing DDE vs. HDE, ^b^ significant difference comparing AgNPs vs. Ag^+^ in AgNO_3_. Values in parenthesis indicate complete formulation concentrations; 96-well plate layouts and supernatant appearance of (**b**) HDE and (**c**) DDE exposed to AgNPs and AgNO_3_ at various concentrations of Ag content.

Figure 2a shows the hemolysis caused by the AgNPs and Ag^+^ from AgNO_3_ on the HDE and DDE. The percentages of hemolysis produced in the HDE by the AgNPs at 12 and 24 µg/mL were 1.96 ± 0.23% and −0.24 ± 0.13%, respectively. Significant differences were found compared with the negative control (*p* ≤ 0.05), but always beneath the limit 5% hemolysis. Regardless of the type of erythrocyte (HDE or DDE), neither of the two hemolytic agents tested seem to trigger any hemolytic mechanism at 12 µg/mL of AgNPs. With 12 µg/mL of silver ions, hemolysis shows no differences from that produced by AgNPs at the same concentration. On the contrary, 24 µg/mL of Ag^+^ has marked hemolysis on the DDE and HDE, with 17.78 ± 3.81 and 13.6 ± 1.9% hemolysis, respectively. Clearly, for both the HDE and DDE the hemolysis caused by AgNPs is much lower than that caused by Ag^+^, with a silver concentration of 24 µg/mL. Comparing the hemolytic effect of AgNPs and Ag^+^ in DDE, we find that the former produces four times less hemolysis (4.2 ± 1.1%) than the latter (17.8 ± 3.8%) at this concentration. Performing the same comparison with the hemolysis values found in the HDE, the sensitivity observed is 68 times greater to Ag^+^ than to the AgNPs, presenting hemolysis values of 13.6 ± 1.9% and −0.24% ± 0.13, respectively. It is important to note that, although the sensitivity of the DDE is higher than for the HDE for AgNPs at 24 µg/mL in relation to hemolysis, in neither case could it be said that the damage is significant since the values are less than the established limit value of 5% (Figure 2a). Thus, AgNPs concentrations of 12 and 24 µg/mL could be considered non-hemolytic.

The concentration of 48 µg/mL from either AgNPs or Ag^+^ produce marked hemolysis, similar to the positive control Triton. Even with marked hemolysis, lower damage by AgNPs is done compared with Ag^+^. AgNPs at 48 µg/mL produce 74.6 ± 5.6% (*** *p* ≤ 0.001) hemolysis, meanwhile Ag^+^ produces 100.3 ± 3.0% (*** *p* ≤ 0.001) on the HDE, a difference that represents 25% of susceptibility for Ag^+^. On the DDE, the difference is close, with 91.8 ± 2.8% (*** *p* ≤ 0.001) hemolysis by AgNPs and 100.3 ± 3% (*** *p* ≤ 0.001) produced by Ag^+^.

Silver ions produce an energy imbalance of erythrocytes even at low concentrations, which in turn leads to eryptosis, a programmed cell death on cells devoid of mitochondria [54]. Thus, the capacity of Ag^+^ release for any AgNPs with a potential biomedical application is a fundamental parameter to be taken into consideration. Only 0.2% of the silver contained in the Argovit™ formulation is released as ions. From a sample of 10 μg/mL AgNPs left under stirring for 24 h, 0.024 μg/mL of Ag^+^ was quantified in solution by inductively coupled plasma atomic emission spectroscopy (ICP-OES) [55]. The larger stability of this formulation that avoids silver ion release is associated with the high amount of polyvinylpyrrolidone (PVP) used as a coating agent [22]. The results presented in this work for hemolysis in HDE (Figure 2a) agree with those reported by Kim [40], who found 4.5, 17.9, and 35.7% hemolysis on erythrocytes exposed to Ag^+^ concentrations of 19, 31.7 and 63.5 µg/mL, respectively. These results confirm that Ag^+^ released from the nanoparticle is much lower than that from dissolved AgNO_3_; thus, Ag^+^ produces greater cytotoxic damage than AgNPs, as other authors have shown with other biomarkers and biological models [2,22,23,56].

The structural changes that hyperglycemia induces in red blood cells, causing osmotic stress and greater fragility in the membrane, as explained in [51,52], contribute to the susceptibility observed. The DDE have a decrease in deformability and are therefore more susceptible to rupture compared to the HDE [40,57]. The difference in structural functionality between the DDE and HDE membranes implies that the former may have a higher percentage of hemolysis than the latter. In summary, for the silver agents studied, hemolysis presents the following sequence:

AgNO_3 DDE_ > AgNO_3 HDE_ > AgNPs _DDE_ > AgNPs _HDE._

Considering the above results of higher fragility of the DDE compared to the HDE, we strongly recommend including not only erythrocytes from healthy donors but also from diabetic donors in future hemolysis studies.

The obtained results of hemolysis in human erythrocytes caused by Argovit™ established a non-hemolytic limit concentration of <24 µg/mL of silver content for both the DDE and HDE. This concentration limit is above the upper concentration of Argovit™, 12 µg/mL, which shows neither cytotoxic nor genotoxic damage on human peripheral blood lymphocytes [22]. The effective antimicrobial and antiviral concentrations are below the non-hemolytic concentrations found in this work [18,58]. The non-hemolytic range found in this work also suggests that concentrations used for biomedical applications, such as the activation of the immunological system [59] or application on the treatment of diabetic foot ulcers [21,50], are far from hemolytic damage.

### Physicochemical Properties and Hemolysis (HDE) by Argovit™ and Other AgNPs Formulations

Due to the lack of data on the silver content for various formulations of AgNPs shown in the Supplementary Material as Table S2, in this section the comparison of our hemolysis results with those reported in the literature is expressed considering the concentration of the complete AgNPs formulation and not that of the Ag content.

**Figure 3:**
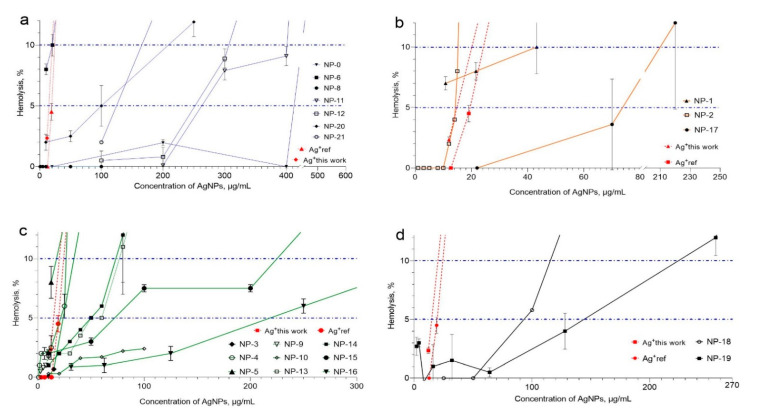
Hemolysis produced by different AgNPs formulations in HDE. AgNP formulations were grouped as follows, considering the coating agents: (**a**) PVP, (**b**) citrate, (**c**) biogenic coating, and (**d**) starch. Label for each AgNPs formulation correspond to label employed in Table S2. For comparative purposes, the limits of no hemolysis (5%) and low hemolysis (10%) are shown with dotted blue horizontal lines.

Hemolysis produced by the AgNP formulations is closely related to the stability provided by the coating agent. Biosynthesized formulations and those coated with citrate present the higher levels of hemolysis, meanwhile starch–AgNPs and PVP formulations produce the lower levels of hemolysis (Table S2 and Figure 3).

Table S2 compiles the physicochemical properties and the hemolytic effects produced by Argovit™ and several AgNP formulations previously reported. For previously reported results, the concentrations were taken as reported in the original source. For Argovit™ AgNPs, both the metallic Ag and PVP contents are provided by the manufacturer. All the AgNP concentrations in Table S2 and Figure 3 correspond to the sum of metallic silver concentration and stabilizer concentration.

The graphical representation of the hemolytic effects, grouped by coating agent, is presented in Figure 3 to facilitate the comparison of data compiled in Table S2. Four groups were formed as follows: nanoparticles stabilized with polyvinylpyrrolidone (PVP), citrate (CIT), biosynthesis coating (BIO), and starch (starch), labeled in Figure 3 with a, b, c, and d, respectively.

The AgNP formulations coated with PVP (Figure 3a) were NP-0 (Argovit™), NP-6, NP-8, NP-11, NP-12, NP-20, and NP-21. As shown in Table S2, these formulations have a spherical shape, with the exception of formulation NP-11, which is a nanowire. The diameters found for these formulations are in the range of 20–100 nm. Figure 3a shows that only NP-0, NP-11 and NP-12 PVP–AgNP formulations with concentrations equal or below 200 µg/mL have no hemolytic effects on the HDE. With concentrations between 200 and 350 µg/mL, the formulations NP-11 and NP-12 show 5–10% hemolysis. With concentrations close to 400 µg/mL, marked hemolysis was observed. On the contrary, NP-0 remains with no hemolytic damage even with concentrations of 400 µg/mL; however, with higher concentrations, marked hemolysis is rapidly produced. The rest of the PVP–AgNP formulations compiled in Table S2 showed more than 5% hemolysis with concentrations lower than 150 µg/mL (Figure 3a). For these AgNPs, 5% hemolysis occurred at concentrations of 15, 100, and 120 µg/mL, respectively.

Comparing the hemolytic effects of spherical PVP–AgNPs, it is found that the formulations NP-12, NP-20, and NP-21 produce more than 5% hemolysis with concentrations equal to or lower than 300 µg/mL; meanwhile, NP-0 requires more than 400 µg/mL. For NP-0, the Ag:PVP ratio is 6:94, while for the other PVP–AgNP formulations (NP-12, NP-20, and NP-21), the reported ratios are 94:6, 99.5:0.2, and 36:64, respectively. Since these formulations are all PVP-based, the only reason we found for the observed difference is the amount of polymer employed to stabilize the AgNPs formulation. The greater the amount of polymer, the greater the stabilization and the lower the release of silver ions, which induces a lower level of observed hemolysis.

A similar argument regarding AgNP stability can be applied to explain the hemolytic damage produced by the other AgNPs with different coating agents, compiled in Table S2. The most unstable citrate–AgNP formulations, compared with PVP–AgNPs, produce more than 5% hemolysis with concentrations lower than 80 µg/mL (NP-1, NP2, and NP-17, Figure 3b). These formulations show diameters between 20 and30 nm by TEM, quite similar to the size of NP-0 (PVP-AgNP, 35 nm); however, their hemolytic potencies are substantially different (Figure 3a,b). NP-1 shows more than 10% hemolysis with a 40 µg/mL concentration, being at least ten times more potent than NP-0. NP-0 produces less than 5% hemolysis at 400 µg/mL. The most dramatic change can be observed with NP-2 (Figure 3b), which produces more than 10% hemolysis with only 15 µg/mL. The hemolytic behavior of NP-2 is practically the same as that observed for silver ions, supporting the proposal that AgNPs stability plays an essential role in their hemolytic potency.

The biogenically produced AgNP formulations show a wide range of hemolysis potency. However, most of the formulations reported have more than 5% hemolysis with concentrations lower than 100 µg/mL, as shown in Figure 3c for NP-3, NP-4, NP-5, NP-13, NP-14, and NP-15. NP-9 was evaluated at a low concentration range but presented a hemolytic tendency similar to that found for NP-3. NP-10 and NP-16 exhibited a lower potency to produce hemolysis among the biogenically produced AgNPs, but concentrations of 250 µg/mL produced more than 5% hemolysis (Figure 3c). The lack of information regarding the molecules that stabilize the formulation limits the possibility to propose a further hypothesis about their hemolytic capacity.

In general, smaller AgNPs produce much more hemolysis than bigger nanoparticles. Figure 3d shows that the starch-stabilized AgNPs have a similar pattern to that observed for the citrated AgNPs; smaller nanoparticles are more hemolytic since NP-18 (≈6 nm) is more hemolytic than NP-19 (17 nm), exceeding 5% hemolysis at 70 and 145 µg/mL, respectively. In the same study, NP-22 coated with polyvinyl alcohol (PVA) (not shown in Figure 3), shows hemolysis higher than 5% at 160 µg/mL, in proximity to NP-19 coated with starch.

NP-0 and NP-21 were specifically compared because these two formulations have the most similar physicochemical properties (Table S2). Both formulations having PVP as stabilizing agent show a similar size (30 and 35 nm). The differences that could be helpful to explain different hemolytic capacities rely on the Ag:PVP ratio and the nature of PVP employed. Besides the stabilization provided by the higher amount of PVP used to produce the AgNP formulations, as discussed on the paragraphs above, the nature of the PVP could contribute to the hemolytic capacity. The molecular mass of PVP in NP-21 is 40 kDa, while in NP-0 it is 12 ± 2.7 kDa. Kyrychenko reported that the higher the molecular mass of PVP, the smaller the surface of the AgNP is covered [60]. When this occurs, the metallic silver surface in the AgNP is more exposed to its surroundings, is more reactive, and can be oxidized faster, making AgNPs less stable. This finding is consistent with the stability reported by the producers of these two formulations of AgNPs. The NanoComposix™ company recommends using its NP-21 formulation in less than 30 days, while the producer Vector-Vita guarantees a stability of its NP-0 for 2 years according to the product label. If the higher molecular mass of PVP in NP-21 leads to faster silver oxidation and higher Ag+ production, these properties could be associated with the much more hemolytic potency of NP-21 compared with NP-0. Therefore, it is not surprising that the 100 nm AgNPs (NP-7) without a coating agent produce 5% hemolysis at approximately 20 µg/mL (Table S2), showing that not stabilized AgNPs are very hemolytic, practically as much as Ag^+^ ions. Thus, the nature and amount of coating agent could play an important role in the hemolytic potency of the AgNP formulations.

According to Shumakova [61], significant symptoms at sub-acute oral toxicity manifested on rats administered with Argovit™, starting from a dose of 1 mg/kg body weight of Ag, and the maximum not observed adverse effect dose (NOAEL) can be estimated as 0.1 mg/kg body weight. In this context, the topical applications of Argovit™ in patients with type II diabetes, which have shown effectiveness against diabetic foot ulcers, resulting in a decrease in edema along with the differentiation of cell lines that promote closure and re-epithelialization of the ulcer [21,50], could be considered within a safe scenario regarding hemolysis. The obtained results of hemolysis produced by Argovit™, in erythrocytes from healthy and diabetic donors, contributes to a deeper understanding of the differential damage elicited by the AgNP formulations related with their toxicological and biocompatibility profiles. Furthermore, these results provide relevant information about the nature and amount of coating agent employed to produce AgNP formulations, factors that must be considered for the design and obtention of safe and effective nanomaterials to be used in the biomedical field.

## 4. Conclusions

The obtained results showed that Argovit™ nanoparticles did not show hemolytic effect at concentrations equal to or lower than 400 µg/mL, in both diabetic and healthy donor erythrocytes. The non-hemolytic concentration range found here is superior to the effective concentration to develop its microbicide, anticancer, and immunostimulating effects. The hemolytic potency of this AgNP formulation is three- to four-times lower compared with the silver ions from AgNO_3_. Therefore, these results let us conclude that the hemolysis observed with more than 400 µg/mL of Argovit™ is not caused by Ag^+^ releasing, but is due to the interaction with the AgNPs as a whole. The maximum non-hemolytic concentration for Argovit™ determined on erythrocytes from healthy and diabetic donors was identified as 400 µg/mL. Argovit™ was found as the least hemolytic formulation for healthy donor erythrocytes compared to 22 other AgNP formulations previously published. The low hemolytic effect was associated with the formulation’s stability, which is finely modulated by the nature and amount of the coating agent employed. Therefore, not only the nature but also the amount of coating agent must be considered in the design and obtention of safe and effective nanomaterials. Finally, this work’s results also show the urgent need to evaluate the hemolytic capacity, among other toxicological parameters, on samples from diabetic donors due to the AgNPs’ widespread use in the treatment of diabetes complications, such as diabetic foot ulcers.

## Figures and Tables

**Table 1:** Concentrations in assay for Ag contained in Argovit™ AgNPs and AgNO_3_.

Tested Substance	(Ag Agent), µg/mL	Silver Content (Ag), µg/mL
AgNPs	200	12
	400	24
	800	48
AgNO_3_	18.86	12
	37.71	24
	75.42	48

## Data Availability

Data are available upon request addressed to the corresponding author.

## Supplementary Materials

The following are available online at https://www.mdpi.com/article/10.3390/ma14112792/s1, Table S1: Statistical analysis, Table S2: AgNPs physicochemical properties and hemolysis results.

## References

[1] Ferrari M. (2005). Cancer nanotechnology: Opportunities and challenges. Nat. Rev. Cancer.

[2] Cameron S.J., Hosseinian F., Willmore W.G. (2018). A Current Overview of the Biological and Cellular Effects of Nanosilver. Int. J. Mol. Sci..

[3] Xu L., Wang Y.-Y., Huang J., Chen C.-Y., Wang Z.-X., Xie H. (2020). Silver nanoparticles: Synthesis, medical applications and biosafety. Theranostics.

[4] Zhang X.-F., Liu Z.-G., Shen W., Gurunathan S. (2016). Silver Nanoparticles: Synthesis, Characterization, Properties, Applications, and Therapeutic Approaches. Int. J. Mol. Sci..

[5] Pareek V., Gupta R., Panwar J. (2018). Do physico-chemical properties of silver nanoparticles decide their interaction with biological media and bactericidal action? A review. Mater. Sci. Eng. C.

[6] Akter M., Sikder T., Rahman M., Ullah A.A., Hossain K.F.B., Banik S., Hosokawa T., Saito T., Kurasaki M. (2018). A systematic review on silver nanoparticles-induced cytotoxicity: Physicochemical properties and perspectives. J. Adv. Res..

[7] Li Y., Qin T., Ingle T., Yan J., He W., Yin J.-J., Chen T. (2016). Differential genotoxicity mechanisms of silver nanoparticles and silver ions. Arch. Toxicol..

[8] Vecchio G., Fenech M., Pompa P.P., Voelcker N.H. (2014). Lab-on-a-Chip-Based High-Throughput Screening of the Genotoxicity of Engineered Nanomaterials. Small.

[9] Ivask A., Voelcker N.H., Seabrook S.A., Hor M., Kirby J.K., Fenech M., Davis T.P., Ke P.C. (2015). DNA Melting and Genotoxicity Induced by Silver Nanoparticles and Graphene. Chem. Res. Toxicol..

[10] Asharani P.V., Mun G.L.K., Hande M.P., Valiyaveettil S. (2009). Cytotoxicity and Genotoxicity of Silver Nanoparticles in Human Cells. ACS Nano.

[11] Foldbjerg R., Jiang X., Miclăuş T., Chen C., Autrup H., Beer C. (2015). Silver nanoparticles—Wolves in sheep’s clothing?. Toxicol. Res..

[12] Guo X., Li Y., Yan J., Ingle T., Jones M.Y., Mei N., Boudreau M.D., Cunningham C.K., Abbas M., Paredes A.M. (2016). Size- and coating-dependent cytotoxicity and genotoxicity of silver nanoparticles evaluated using in vitro standard assays. Nanotoxicology.

[13] Butler K.S., Peeler D.J., Casey B.J., Dair B.J., Elespuru R.K. (2015). Silver nanoparticles: Correlating nanoparticle size and cellular uptake with genotoxicity. Mutagenesis.

[14] Milić M., Leitinger G., Pavičić I., Avdičević M.Z., Dobrović S., Goessler W., Vrček I.V. (2015). Cellular uptake and toxicity effects of silver nanoparticles in mammalian kidney cells. J. Appl. Toxicol..

[15] Ahmed L.B., Milić M., Pongrac I.M., Marjanović A.M., Mlinarić H., Pavičić I., Gajović S., Vrček I.V. (2017). Impact of surface functionalization on the uptake mechanism and toxicity effects of silver nanoparticles in HepG2 cells. Food Chem. Toxicol..

[16] Franchi L.P., Manshian B.B., de Souza T.A., Soenen S., Matsubara E.Y., Rosolen J.M., Takahashi C.S. (2015). Cyto- and genotoxic effects of metallic nanoparticles in untransformed human fibroblast. Toxicol. Vitr..

[17] Bravo-Guerra C., Cáceres-Martínez J., Vásquez-Yeomans R., Pestryakov A., Bogdanchikova N. (2020). Lethal effects of silver nanoparticles on Perkinsus marinus, a protozoan oyster parasite. J. Invertebr. Pathol..

[18] Vazquez-Muñoz R., Meza-Villezcas A., Fournier P., Soria-Castro E., Juarez-Moreno K., Gallego-Hernández A.L., Bogdanchikova N., Vazquez-Duhalt R., Huerta-Saquero A. (2019). Enhancement of antibiotics antimicrobial activity due to the silver nanoparticles impact on the cell membrane. PLoS ONE.

[19] Valenzuela-Salas L.M., Girón-Vázquez N.G., García-Ramos J.C., Torres-Bugarín O., Gómez C., Pestryakov A., Villarreal-Gómez L.J., Toledano-Magaña Y., Bogdanchikova N. (2019). Antiproliferative and Antitumour Effect of Nongenotoxic Silver Nanoparticles on Melanoma Models. Oxidative Med. Cell. Longev..

[20] Guerra J.D., Sandoval G., Avalos-Borja M., Pestryakov A., Garibo D., Susarrey-Arce A., Bogdanchikova N., Patron A. (2020). Selective antifungal activity of silver nanoparticles: A comparative study between Candida tropicalis and Saccharomyces boulardii. Colloid Interface Sci. Commun..

[21] Hernández C.A.A., Clinic E.I.A.A., Juarez-Moreno K., Castañeda-Juarez M.E., Almanza-Reyes H., Pestryakov A., Bogdanchikova N. (2017). Silver Nanoparticles for the Rapid Healing of Diabetic Foot Ulcers. Int. J. Med. Nano Res..

[22] Ruiz-Ruiz B., Arellano-García M.E., Radilla-Chávez P., Salas-Vargas D.S., Toledano-Magaña Y., Casillas-Figueroa F., Vazquez-Gomez R.L., Pestryakov A., García-Ramos J.C., Bogdanchikova N. (2020). Cytokinesis-Block Micronucleus Assay Using Human Lymphocytes as a Sensitive Tool for Cytotoxicity/Genotoxicity Evaluation of AgNPs. ACS Omega.

[23] Casillas-Figueroa F., Arellano-García M.E., Leyva-Aguilera C., Ruíz-Ruíz B., Vázquez-Gómez R.L., Radilla-Chávez P., Chávez-Santoscoy R.A., Pestryakov A., Toledano-Magaña Y., García-Ramos J.C. (2020). Argovit™ Silver Nanoparticles Effects on *Allium cepa*: Plant Growth Promotion without Cyto Genotoxic Damage. Nanomaterials.

[24] Platonova T.A., Pridvorova S.M., Zherdev A.V., Vasilevskaya L.S., Arianova E.A., Gmoshinski I.V., Khotimchenko S.A., Dzantiev B.B., Popov V.O., Tutelyan V.A. (2013). Identification of silver nanoparticles in the small intestinal mucosa, liver, and spleen of rats by transmission electron microscopy. Bull. Exp. Biol. Med..

[25] Buzulukov Y.P., Arianova E.A., Demin V.F., Safenkova I.V., Gmoshinski I.V., Tutelyan V.A. (2014). Bioaccumulation of silver and gold nanoparticles in organs and tissues of rats studied by neutron activation analysis. Biol. Bull..

[26] Gmoshinski I.V., Khotimchenko S.A., Popov V.O., Dzantiev B.B., Zherdev A.V., Demin V.F., Buzulukov Y.P. (2013). Nanomaterials and nanotechnologies: Methods of analysis and control. Russ. Chem. Rev..

[27] Bernauer U., Bodin L., Chaudhry Q., Coenraads P.J., Dusinska M., Gaffet E., Panteri E., Rogiers V., Rousselle C., Stepnik M. (2020). The SCCS guidance on the safety assessment of nanomaterials in cosmetics. Regul. Toxicol. Pharmacol..

[28] ASTM (2013). Standard Test Method for Analysis of Hemolytic Properties of Nanoparticles.

[29] Sowemimo-Coker S.O. (2002). Red blood cell hemolysis during processing. Transfus. Med. Rev..

[30] Weed R.I., Reed C.F., Berg G. (1963). Is hemoglobin an essential structural component of human erythrocyte membranes?. J. Clin. Investig..

[31] Huang H., Lai W., Cui M., Liang L., Lin Y., Fang Q., Liu Y., Xie L. (2016). An Evaluation of Blood Compatibility of Silver Nanoparticles. Sci. Rep..

[32] Chi Z., Lin H., Li W., Zhang X., Zhang Q. (2018). In vitro assessment of the toxicity of small silver nanoparticles and silver ions to the red blood cells. Environ. Sci. Pollut. Res..

[33] Sen I.K., Mandal A.K., Chakraborti S., Dey B., Chakraborty R., Islam S.S. (2013). Green synthesis of silver nanoparticles using glucan from mushroom and study of antibacterial activity. Int. J. Biol. Macromol..

[34] Halbandge S.D., Mortale S.P., Karuppayil S.M. (2017). Biofabricated Silver Nanoparticles Synergistically Activate Amphotericin B Against Mature Biofilm Forms of Candida Albicans. Open Nanomed. J..

[35] Nasar M.Q., Zohra T., Khalil A.T., Saqib S., Ayaz M., Ahmad A., Shinwari Z.K. (2019). Seripheidium quettense mediated green synthesis of biogenic silver nanoparticles and their theranostic applications. Green Chem. Lett. Rev..

[36] Choi J., Reipa V., Hitchins V.M., Goering P.L., Malinauskas R.A. (2011). Physicochemical Characterization and In Vitro Hemolysis Evaluation of Silver Nanoparticles. Toxicol. Sci..

[37] Laloy J., Minet V., Alpan L., Mullier F., Beken S., Toussaint O., Lucas S., Dogné J.-M. (2014). Impact of Silver Nanoparticles on Haemolysis, Platelet Function and Coagulation. Nanobiomedicine.

[38] Hamouda R.A., Hussein M.H., Abo-Elmagd R.A., Bawazir S.S. (2019). Synthesis and biological characterization of silver nanoparticles derived from the cyanobacterium *Oscillatoria limnetica*. Sci. Rep..

[39] Katva S., Das S., Moti H.S., Jyoti A., Kaushik S. (2018). Antibacterial Synergy of Silver Nanoparticles with Gentamicin and Chloramphenicol against Enterococcus faecalis. Pharmacogn. Mag..

[40] Kim M.J., Shin S. (2014). Toxic effects of silver nanoparticles and nanowires on erythrocyte rheology. Food Chem. Toxicol..

[41] Hossain M., Polash S.A., Takikawa M., Shubhra R.D., Saha T., Islam Z., Hossain S., Hasan A., Takeoka S., Sarker S.R. (2019). Investigation of the Antibacterial Activity and in vivo Cytotoxicity of Biogenic Silver Nanoparticles as Potent Therapeutics. Front. Bioeng. Biotechnol..

[42] Shah A., Lutfullah G., Ahmad K., Khalil A.T., Maaza M. (2018). Daphne mucronata-mediated phytosynthesis of silver nanoparticles and their novel biological applications, compatibility and toxicity studies. Green Chem. Lett. Rev..

[43] Parthiban E., Manivannan N., Ramanibai R., Mathivanan N. (2019). Green synthesis of silver-nanoparticles from Annona reticulata leaves aqueous extract and its mosquito larvicidal and anti-microbial activity on human pathogens. Biotechnol. Rep..

[44] Asharani P.V., Sethu S., Vadukumpully S., Zhong S., Lim C.T., Hande M.P., Valiyaveettil S. (2010). Investigations on the Structural Damage in Human Erythrocytes Exposed to Silver, Gold, and Platinum Nanoparticles. Adv. Funct. Mater..

[45] Siritongsuk P., Hongsing N., Thammawithan S., Daduang S., Klaynongsruang S., Tuanyok A., Patramanon R. (2016). Two-Phase Bactericidal Mechanism of Silver Nanoparticles against Burkholderia pseudomallei. PLoS ONE.

[46] Bian Y., Kim K., Ngo T., Kim I., Bae O.-N., Lim K.-M., Chung J.-H. (2019). Silver nanoparticles promote procoagulant activity of red blood cells: A potential risk of thrombosis in susceptible population. Part. Fibre Toxicol..

[47] Paladini F., Pollini M. (2019). Antimicrobial Silver Nanoparticles for Wound Healing Application: Progress and Future Trends. Materials.

[48] Ezhilarasu H., Vishalli D., Dheen S.T., Bay B.-H., Srinivasan D.K. (2020). Nanoparticle-Based Therapeutic Approach for Diabetic Wound Healing. Nanomaterials.

[49] Li Z. (2017). Preparation of High Valence Silver Complex Nanoparticles for Diabetic Foot Ulcers Application. Master’s Thesis.

[50] Almonaci-Hernández C.A., Luna-Vazquez-Gomez R., Luna-Vazquez-Gomez R.A., Valenciano-Vega J.I., Carriquiry-Chequer N.I., Rembao-Hernández A., Gomez-Zendejas M.L., Almanza-Reyes H., Garibo-Ruiz D., Pestryakov A. (2020). Nanomedicine Approach for the Rapid Healing of Diabetic Foot Ulcers with Silver Nanoparticles. J. Clin. Med Images.

[51] Priyadarshini K.H., Latha P.A., Pradnya S., Juhi A., Samatha P., Ratnam K.M. (2015). Comparative study of erythrocyte fragility in diabetes mellitus and non diabetes mellitus. Int. J. Med. Res. Health Sci..

[52] Rafiq S., Rownak N., Akhter S., Khatun M., Baksh S., Rahman M. (2017). Study of Osmotic Fragility Status of Red Blood Cell in Type II Diabetes Mellitus Patients. Eur. J. Environ. Public Health.

[53] Stephano-Hornedo J.L., Torres-Gutiérrez O., Toledano-Magaña Y., Gradilla-Martínez I., Pestryakov A., Sánchez-González A., García-Ramos J.C., Bogdanchikova N. (2020). Argovit™ silver nanoparticles to fight Huanglongbing disease in Mexican limes (*Citrus aurantifolia* Swingle). RSC Adv..

[54] Sopjani M., Föller M., Haendeler J., Götz F., Lang F. (2009). Silver ion-induced suicidal erythrocyte death. J. Appl. Toxicol..

[55] Vazquez-Muñoz R., Bogdanchikova N., Huerta-Saquero A. (2020). Beyond the Nanomaterials Approach: Influence of Culture Conditions on the Stability and Antimicrobial Activity of Silver Nanoparticles. ACS Omega.

[56] Nallanthighal S., Chan C., Bharali D.J., Mousa S., Vásquez E., Reliene R. (2017). Particle coatings but not silver ions mediate genotoxicity of ingested silver nanoparticles in a mouse model. NanoImpact.

[57] Ibanga I.A., Usoro C.A., Nsonwu A.C. (2005). Glycaemic control in type 2 diabetics and the mean corpuscular fragility. Niger. J. Med..

[58] Borrego B., Lorenzo G., Mota-Morales J.D., Almanza-Reyes H., Mateos F., López-Gil E., de la Losa N., Burmistrov V.A., Pestryakov A.N., Brun A. (2016). Potential application of silver nanoparticles to control the infectivity of Rift Valley fever virus in vitro and in vivo. Nanomed. Nanotechnol. Biol. Med..

[59] Castro-Gamboa S., Garcia-Garcia M.R., Piñon-Zarate G., Rojas-Lemus M., Jarquin-Yañez K., Herrera-Enriquez M.A., Fortoul T.I., Toledano-Magaña Y., Garcia-Iglesias T., Pestryakov A. (2019). Toxicity of silver nanoparticles in mouse bone marrow-derived dendritic cells: Implications for phenotype. J. Immunotoxicol..

[60] Kyrychenko A., Korsun O.M., Gubin I.I., Kovalenko S.M., Kalugin O.N. (2015). Atomistic Simulations of Coating of Silver Nanoparticles with Poly(vinylpyrrolidone) Oligomers: Effect of Oligomer Chain Length. J. Phys. Chem. C.

[61] Shumakova A.A., Shipelin V.A., Efimochkina N.R., Minaeva L.P., Bykova I.B., Markova Y.M., Trushina E.N., Mustafina O.K., Gmoshinsky I.V., Khanferyan R.A. (2016). Toxicological evaluation of colloidal nano-sized silver stabilized polyvinylpyrrolidone. IV. Influence on intestinal microbiota, immune indexes. Voprosy Pitaniia.

